# Peri-acetabular bone remodelling after uncemented total hip arthroplasty with monoblock press-fit cups: an observational study

**DOI:** 10.1186/s12891-020-03675-7

**Published:** 2020-10-06

**Authors:** Conrad Anderl, Georg Mattiassich, Reinhold Ortmaier, Martin Steinmair, Josef Hochreiter

**Affiliations:** 1 Department of Orthopaedic Surgery, Ordensklinikum Barmherzige Schwestern, Vinzenz Gruppe, Center of Orthopaedic Excellence, Teaching Hospital of the Paracelsus Medical University Salzburg, Seilerstätte 4, 4020 Linz, Austria; 2 Ludwig-Boltzmann Institute for Experimental and Clinical Traumatology, Donaueschingenstrasse 13, 1200 Vienna, Austria; 3 Research Unit of Orthopedic Sports Medicine and Injury Prevention, Institute for Sports Medicine, Alpine Medicine and Health Tourism (ISAG), UMIT, Eduard-Wallnöfer-Zentrum 1, 6060 Hall in Tirol, Austria

**Keywords:** Total hip arthroplasty, Monoblock cups, Bone remodelling, Elastic fixation, Bone mineral density, DEXA

## Abstract

**Background:**

Bone stock preservation in total hip replacement is essential to allow for later revisions in an increasingly younger and fitter index patient population. While contemporary modular press-fit acetabular cups lead to rigid fixation with equatorial stress and central osteolysis, more elastic fixation may cause different peri-acetabular bone remodelling. The purpose of this study was to investigate changes in peri-acetabular bone mineral density (BMD) in uncemented elastic fixation with monoblock press-fit cups.

**Methods:**

This prospective observational study included 45 patients with monoblock cups. We evaluated peri-acetabular BMD using dual-energy X-ray absorptiometry and reported functional outcomes and complications.

**Results:**

At a mean follow-up of 24.2 ± 2.2 months, we found that BMD stabilised in DeLee and Charnley zones I and III and recovered to baseline value in zone II. The mean Harris Hip Scores improved significantly from 56.9 ± 20.0 to 97.2 ± 4.0 (*p* <  0001). Other than one peri-operative dislocation, we saw no post-operative complications.

**Conclusions:**

We found favourable adaptive bone changes with BMD stabilisation in the equatorial zones and recovery to pre-operative values in the central zone. Additionally, excellent clinical outcomes and few prosthesis-related complications strengthened the favourable results of monoblock acetabular cups.

**Trial registration:**

Registration number DRKS00017076.

## Background

Initially, total hip arthroplasty (THA) was primarily used in low-demand elderly patients. As prosthetic design developed, however, indications for THA expanded to include younger and more active patients with higher demands [1]. Because younger patients were more at risk to undergo revision surgeries in the future [2], treating bone stock and soft tissue with care at the index procedure became increasingly more important to allow future revisions [3].

Revision of the acetabular component is a limiting factor in the longevity of hip replacement with wear and aseptic loosening being leading causes of failure [4–6]. Primary fixation of the cup is therefore extremely important to achieve long-lasting results.

Two types of biomechanical fixation concepts may be employed in acetabular fixation of uncemented THA depending on cup design: rigid or elastic fixation. Contemporary modular press-fit acetabular cups have stiffer metal shells than monoblock press-fit cups, which result in rigid fixation. Such a fixation is known to transmit forces in the equatorial region (DeLee and Charnley zones I and III), increasing stress concentrations around the acetabular rim and reducing stress within the bone proximal to the implant (DeLee and Charnley zone II) [7, 8]. In fact, finite element analysis and computer-simulated modelling have shown that changes in peri-acetabular bone mineral density (BMD) occur in response to altered pelvic stress patterns [5]. Over time, BMD changes translate into increased central osteolysis around stiff metal-backed cups eventually leading to loosening [7, 8], one of the leading causes of failure of the acetabular component after primary THA [4, 5].

Monoblock press-fit cups, on the other hand, are less stiff [7]. Consequently, fixation of these cups is expected to result in less equatorial stress and less central osteolysis [7]. However, little clinical evidence exists to support this fixation concept [7, 9].

Therefore, in this prospective observational study, we examined bone remodelling around a monoblock acetabular cup using dual-energy X-ray absorptiometry (DEXA). We hypothesised that, owing to elastic fixation, bone loss around this prosthesis would be less dramatic in the central acetabular zone than the equatorial zones. We also assessed functional outcomes and complications.

## Methods

### Study setup

This was a single-centre prospective observational study performed at the Department of Orthopaedic Surgery of the Ordensklinikum Barmherzige Schwestern in Linz, Austria. Patients with osteoarthritis treated with THA were enrolled consecutively at a single institution in Austria from November 2014 to August 2015. Due to the inherently altered biomechanics and the presumed effect on osteolysis, we excluded patients who had undergone previous surgery of the affected hip, had received arthroplasty for other joints of the lower limbs, required bilateral THA, suffered from relevant comorbidities, or were either unable or unwilling to participate in the study. Clinical and radiographic examinations were carried out pre-operatively, and three, 12, and 24 months post-operatively.

The study protocol was approved by the local ethics committee. The institutional review board also approved the study (ethics approval registration number: EK 19/14; issue date: 16 June 2014). The study was also registered in the German Clinical Trials Register (clinical trial registration number: DRKS00017076). We conducted the study in accordance with the study protocol, the latest Helsinki Declaration, and good clinical practice guidelines.

### Surgical technique, implant design, and post-operative rehabilitation

All patients received spinal or general anaesthesia and were placed in a supine position. The surgeries were performed by five senior orthopaedic surgeons, using an anterolateral, muscle-preserving approach between the tensor fasciae latae and the gluteus medius muscles.

All patients underwent uncemented short-stem THA. On the acetabular side, they received a monoblock press-fit acetabular cup (RM Pressfit vitamys; Mathys Ltd. Bettlach, Switzerland) made of, highly cross-linked, vitamin E-infused polyethylene. The cup achieves primary stability by equatorial press-fit and secondary stability by bony on-growth to the titanium coating. The coating itself conveys no structural stiffness, which allows the cup to remain isoelastic. On the femoral side, they received a calcar-guided femoral short-stem prosthesis with a titanium plasma spray and calcium phosphate coating (optimys Stem; Mathys Ltd. Bettlach, Switzerland) combined with ceramic femoral heads (ceramys Hip Head; Mathys Ltd. Bettlach, Switzerland) of 28 mm in five and 32 mm in the remaining patients.

On the first post-operative day, patients began full weight-bearing under the supervision of a physiotherapist. Active and passive mobilisation with restricted joint flexion was encouraged. Following discharge, patients either underwent outpatient physiotherapy or were transferred to inpatient rehabilitation centres.

### Dual-energy X-ray absorptiometry

**Fig. 1:**
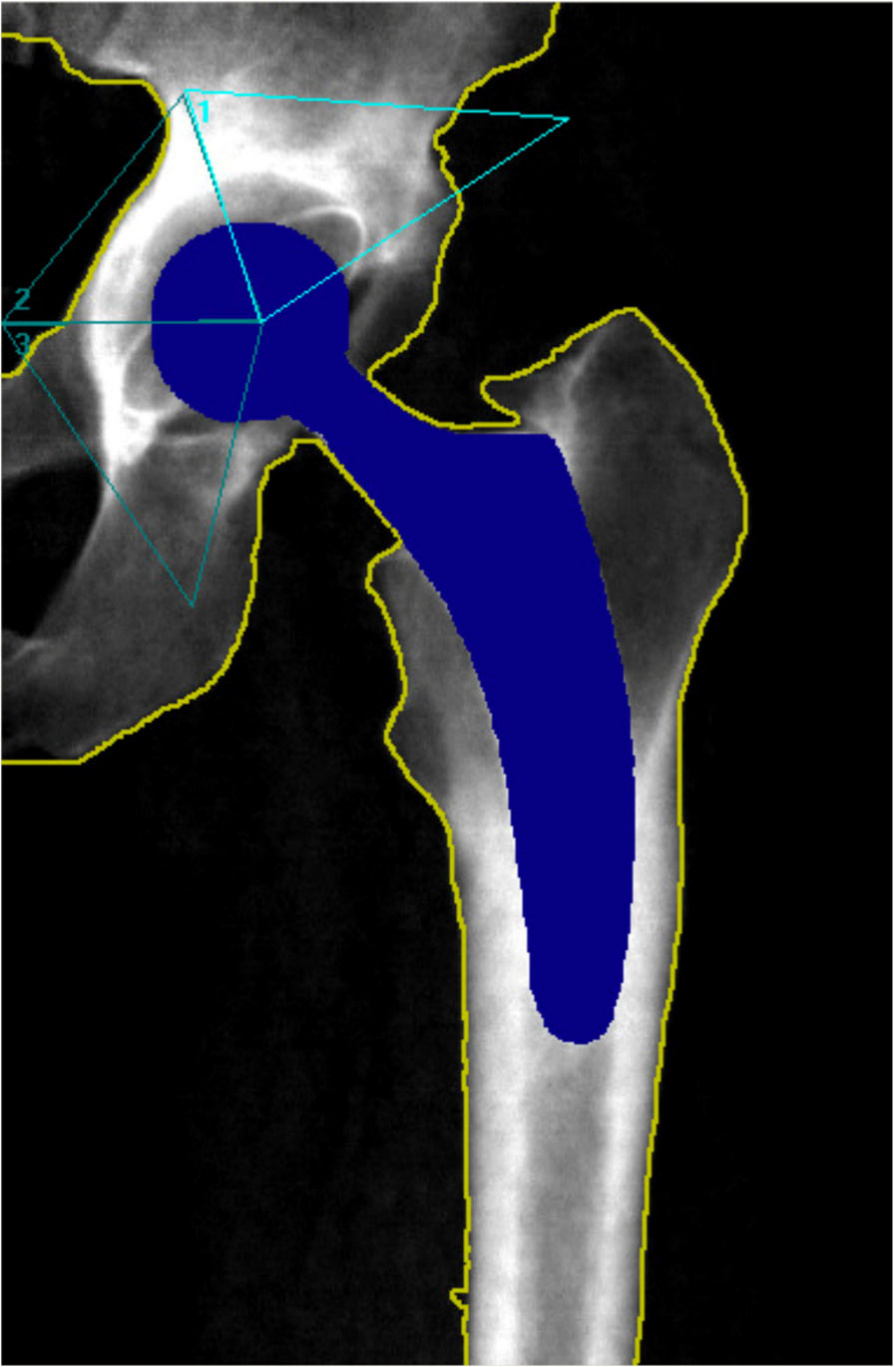
Dual-energy X-ray absorptiometry image of the prosthesis with modified DeLee and Charnley zones

We measured BMD around the prosthesis pre-operatively (baseline) and at three, 12, and 24 months post-operatively using the bone densitometer Lunar iDXA (GE Healthcare Lunar, Madison, Wisconsin, USA). We recorded absolute BMD values in three regions of interest defined according to a modified DeLee and Charnley model (Fig. 1). Additionally, we calculated the BMD change in each zone by dividing the measured BMD by the baseline value and expressed the ratio as a percentage.

We measured BMD with patients in a supine position and the operated leg internally rotated 20°, which allowed us to prevent errors in measurement [10]. A blinded investigator analysed all DEXA measurements. This investigator did neither participate in the surgeries or in post-operative patient care, nor did he have access to study data.

### Radiographic and clinical assessment

Pre- and post-operative radiographic examinations included a standing anteroposterior radiograph of the pelvis and an axial radiograph of the operated hip. Follow-up examinations included a anteroposterior and axial radiographs only of the affected hip. Standardised templates, as described by DeLee and Charnley [11], were used to locate peri-prosthetic abnormalities and bone loss.

Acetabular cup inclination angles were measured post-operatively from the anteroposterior radiographs and differentiated into three categories: < 40°, between 40° and 50°, and > 50°.

Harris Hip Score (HHS) was used to evaluate range of motion, pain, and function. The questionnaire included a visual analogue scale (VAS) from zero to ten to assess pain at rest and under load, with zero representing no pain and ten the worst pain. We also asked patients to rate their overall satisfaction on a scale of zero to ten, with zero representing the lowest and ten the highest level of patient satisfaction.

### Statistical analysis

Descriptive statistics included means, medians, standard deviations, and ranges. Sample size estimation was carried out for the femoral components only. We estimated that a sample size of 45 patients would be sufficient to detect relative differences of 17% or more with a power of 99.7% based on a standard deviation of the difference of 0.26 [12]. We used paired t tests to evaluate BMD differences to baseline. The level of significance was set at 0.05 (two-sided) for all tests. All statistical analyses were performed with SAS version 9.4 (SAS Institute Inc., Cary, North Carolina, USA).

## Results

### Study participants

**Fig. 2:**
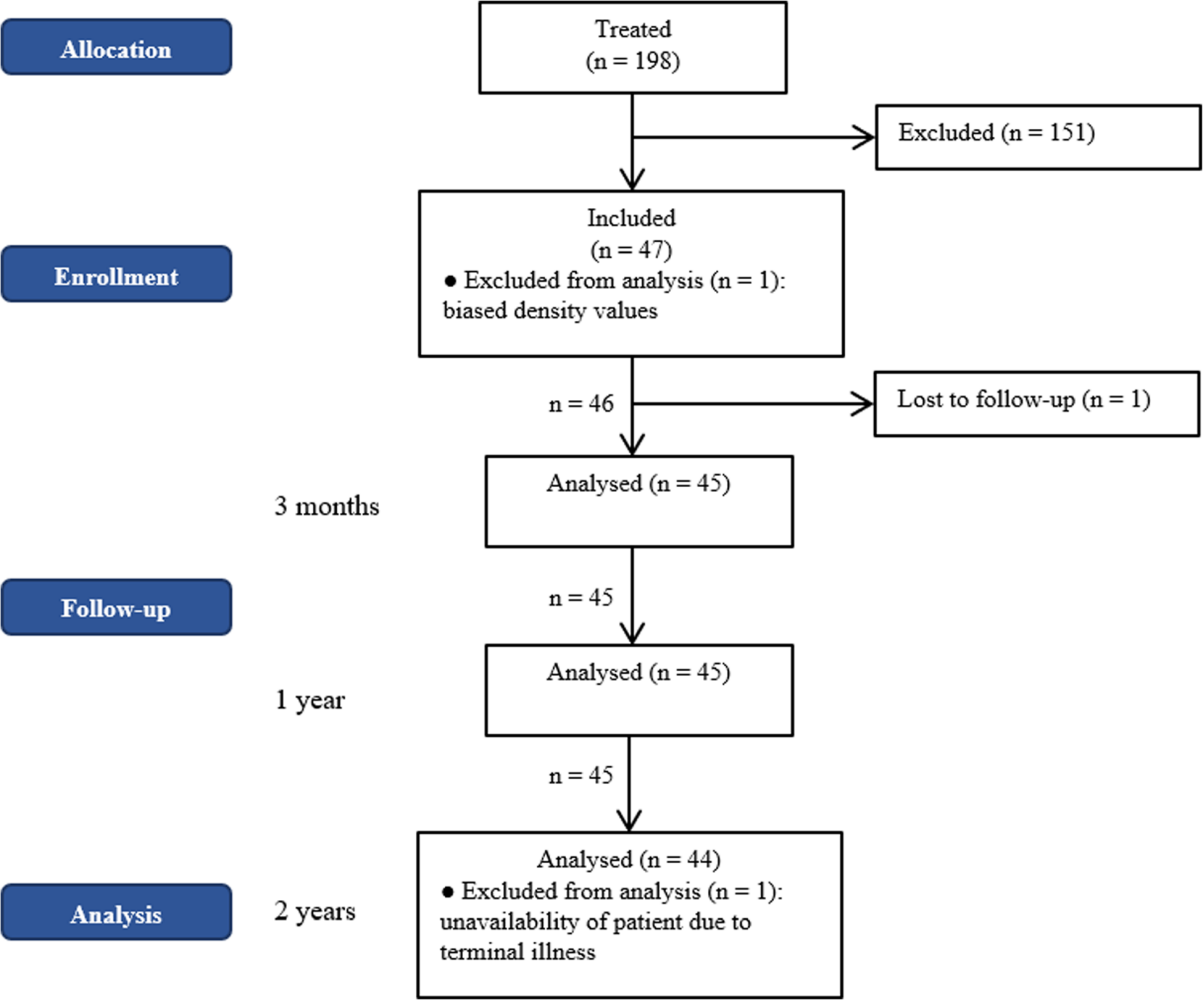
Concise patient flowchart of allocation, enrolment, follow-up, and analysis

In total, 198 consecutive patients were treated with the monoblock press-fit cup (Fig. 2). Overall, 151 patients did not meet the inclusion criteria or were unable or unwilling to participate in the study, leaving 47 patients for analysis. Of these, one patient was lost to follow-up and another was excluded due to severe sclerosis resulting in overly biased density values. This patient had pre-operative BMD values at the cup region around four times higher than the second highest value or eight times higher than the median value of the study population. This left 45 patients for analysis, 44 of which completed the 24-month follow-up examination; one patient missed the last follow-up examination due to terminal illness.

Patients were followed for a mean of 24.2 ± 2.2 months. They had a mean age of 65.5 ± 9.4 years at the time of surgery and a male-to-female ratio of 21:24.

### Dual-energy X-ray absorptiometry

**Fig. 3:**
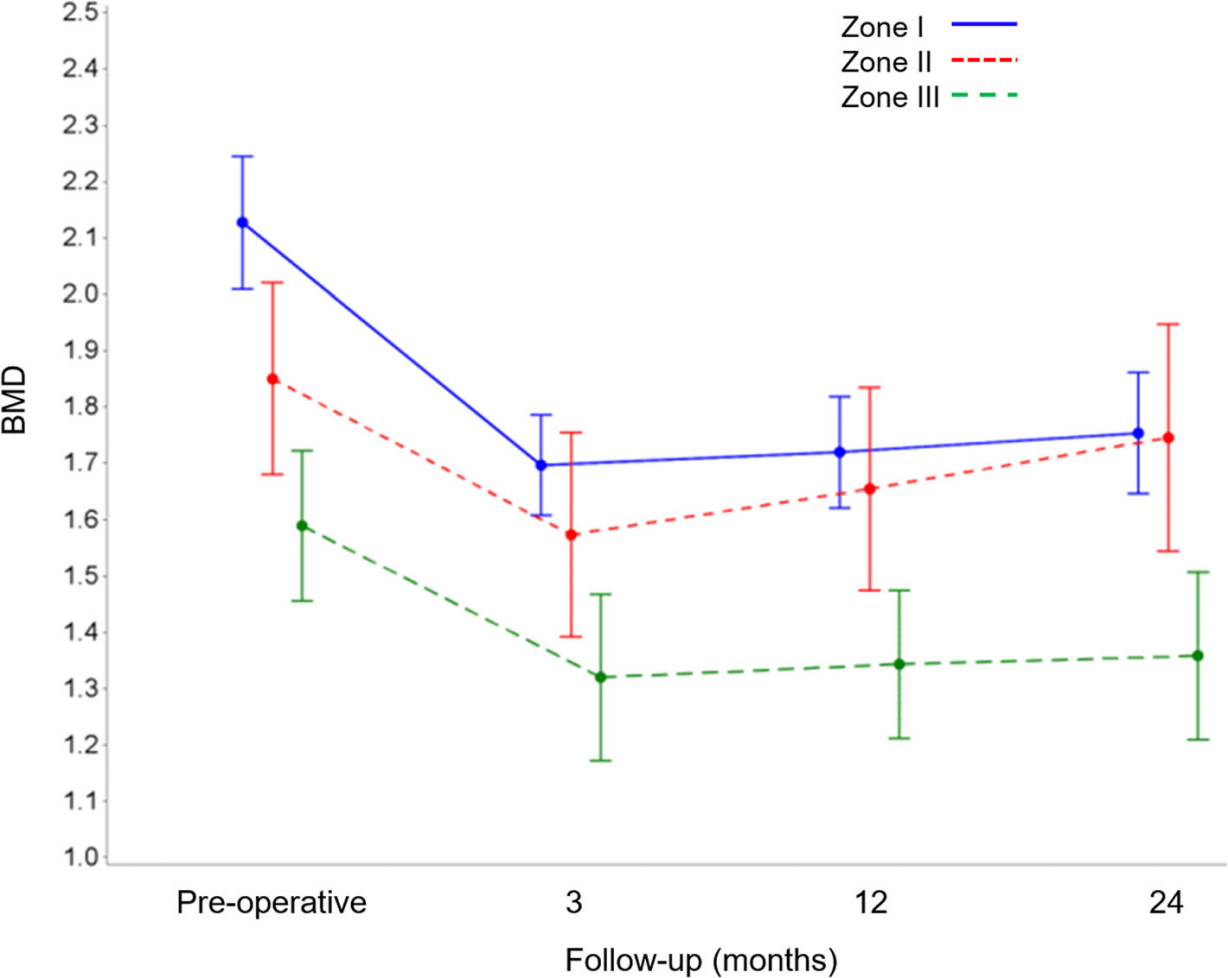
Mean changes in bone mineral density from baseline by modified DeLee and Charnley zone and follow-up time point. BMD: bone mineral density

We observed a significant initial decrease in BMD in all zones three months post-operatively compared with baseline values (*p* = 0.013) (Fig. 3). At later follow-up time points, we found that BMD had stabilised in zones I and III and had recovered in zone II to such an extent that it was no longer significantly different from the baseline value (*p* = 0.171), indicating a nearly complete BMD recovery in zone II (Table 1).

**Table 1:** Absolute and relative changes of bone mineral density by modified DeLee and Charnley zone and follow-up time point, expressed as means (standard deviations)

DeLee and Charnley zone	3 months		12 months		24 months		***P*** value^a^
	Absolute change (g/cm^2^)	Relative change (%)	Absolute change (g/cm^2^)	Relative change (%)	Absolute change (g/cm^2^)	Relative change (%)	
I	−0.4 (0.3)	−19.0 (9.0)	−0.4 (0.3)	−18.0 (13.0)	− 0.4 (0.3)	−17.1 (11.0)	< 0.0001
II	−0.3 (0.4)	−13.1 (19.0)	− 0.2 (0.6)	− 8.0 (30.0)	− 0.1 (0.6)	−4.0 (29.0)	0.171
III	− 0.3 (0.1)	−17.0 (9.0)	− 0.3 (0.2)	−16.0 (10.3)	− 0.2 (0.3)	− 15.1 (17.0)	< 0.0001

^a^Difference to baseline at 24-month follow-up examination

### Radiographic and clinical outcomes

The mean post-operative cup inclination angle was 43.9° ± 5.7°. The mean HHS improved significantly: from 56.9 ± 19.9 pre-operatively to 97.2 ± 4.0 at 24 months post-operatively (*p* <  0.0001) (Table 2). At the final follow-up examination, all other scores had also significantly improved compared to the baseline. The mean VAS for pain at rest decreased by 4.4 points (*p* <  0.0001), the mean VAS for pain while weight-bearing decreased by 6.7 points (*p* <  0.0001), and the VAS for satisfaction increased by 7.7 points (*p* <  0.0001).

**Table 2:** Clinical outcomes by follow-up time point, expressed as mean (standard deviation)

Clinical Outcome		Pre-operative	3 months	12 months	24 months	***p*** value^a^
**HHS**		56.9 (19.9)	94.2 (6.1)	95.4 (5.5)	97.2 (4.0)	< 0.0001
**VAS for pain**	**at rest**	4.5 (2.9)	0.5 (1.7)	0.3 (1.3)	0.2 (1.2)	< 0.0001
	**under load**	7.1 (1.9)	0.9 (1.1)	0.4 (1.1)	0.4 (1.1)	< 0.0001
**VAS for satisfaction**		2.0 (2.0)	9.4 (0.7)	9.6 (1.1)	9.7 (1.1)	< 0.0001

^a^Difference to baseline at 24-month follow-up examination. HHS: Harris Hip Score, VAS: visual analogue scale

### Complications

One patient experienced a dislocation during patient repositioning from the operating table to the hospital bed after surgery, which was treated immediately with closed reduction. During the observation period no post-operative complications were reported, and also no revision surgeries were required.

## Discussion

Aim of this study was to evaluated bone remodelling around an uncemented monoblock acetabular cup. We hypothesised that the acetabular cup would distribute stress equally, thereby mitigating disproportionate central zone resorption. However, favourable adaptive changes could be observed during the follow-up period with the greatest BMD recovery seen in zone II.

In a recent prospective study examining rigid fixation with a modular press-fit cup, the authors found an initial decrease, followed by recovery of BMD in all three zones at 24 month of follow-up [5]. Although BMD did recover and stabilise in all zones, it remained significantly lower at follow-up than the baseline values [5]. Additionally, osteoconductive coating, such as hydroxyapatite [13] or alumina-reduced surface finish [14], did not improve peri-acetabular bone remodelling around modular cups. These data suggest that rigid acetabular fixation results in a loss of BMD in all zones over time and that this loss cannot be recovered by increasing the osteointegration properties of modular acetabular cups.

In contrast, a recent clinical study involving texture analysis of elastic fixation with a monoblock press-fit acetabular cup showed statistically significant changes in peri-acetabular stress distribution with an increase in peri-prosthetic bone in zone II up to five years post-operatively [7]. A second study involving the same monoblock press-fit acetabular cup found both a homogeneous appearance of the bone trabeculae around the cup and resolution of pre-operative sclerosis one and a half years post-operatively, suggesting that the implant distributed loads more evenly [9]. Our study also revealed similar results with a monoblock press-fit acetabular cup made of vitamin E-infused highly cross-linked polyethylene using DEXA. Taken together, these results strengthen the concept that elastic fixation leads to a more even distribution of stress around the acetabulum resulting in better bone recovery in zone II than rigid fixation. Preservation of bone is important, especially for THA in young patients, who are likely to undergo revision surgery later in life [2].

With regard to clinical results, functional outcomes measured by the HHS improved substantially over the first three months and remained favourable during follow-up (mean HHS of 97.2). Similarly high values have been reported in other recent studies with longer follow-up periods [15, 16]. Furthermore, we observed no complications except one peri-operative dislocation, as aforementioned. Specifically, there were no cases of post-operative dislocation or peri-prosthetic fracture, both of which are commonly encountered after conventional uncemented THA [17, 18]. A recent study with the same acetabular cup as used in our study found one case (1%) of aseptic loosening after a mean follow-up of 4.75 years; however, they attributed this to the young patient population (mean age of 55.2 years) enrolled in their study [16]. At 24 months, we saw no cases of aseptic loosening. Another study with monoblock acetabular cups found no cases of aseptic loosening even at a mean follow-up of 15.6 years, confirming the low rates of long-term THA complications such as migration, loosening, and wear with monoblock acetabular cups [15].

Our study has several strengths. It was a prospective study with consecutive patient enrolment, yielding an uninterrupted and complete dataset. Additionally, DEXA measurements are considered reliable and not prone to subjective bias, making our results comparable with other studies [19]. Nevertheless, this study does have some weaknesses. First, patient follow-up was limited to 24 months. Longer follow-up periods may be necessary to confirm these findings. Second, our study lacked a control group. Direct comparison of monoblock and modular cups might have revealed a clearer picture of the advantages and disadvantages of these implants. Finally, although DEXA is currently the most widely used technique to measure BMD after THA [5], it does not provide three-dimensional information on the exact bone distribution.

## Conclusions

In conclusion, we demonstrated that favourable adaptive peri-acetabular bone changes occur using a monoblock acetabular cup. After an initial decrease, BMD stabilised in the equatorial zones and recovered in the central zone to pre-operative values. Additionally, we saw excellent clinical outcomes and encountered few prosthesis-related complications, strengthening the good results of monoblock acetabular cups.

## Data Availability

All data generated or analysed during this study are included in this article.

## Abbreviations

BMD: bone mineral density
THA: total hip arthroplasty
DEXA: dual-energy X-ray absorptiometry
HHS: Harris Hip Score
VAS: visual analogue scale
